# Acute hyperactive delirium-like episode during dexmedetomidine sedation after recent discontinuation of long-term reserpine therapy: a case report

**DOI:** 10.3389/fmed.2026.1894698

**Published:** 2026-07-29

**Authors:** Baohua Zhang, Yang Han, Lin Zong, Lidong Zhang

**Affiliations:** 1 Department of Anesthesiology, Jinling Hospital, Affiliated with the Medical School of Nanjing University, Nanjing, Jiangsu, China; 2 Department of Pain Medicine, Jinling Hospital, Affiliated with the Medical School of Nanjing University, Nanjing, Jiangsu, China

**Keywords:** case report, delirium-like episode, dexmedetomidine, reserpine, spinal anesthesia

## Abstract

**Background:**

Dexmedetomidine is a commonly used perioperative sedative and is generally associated with a lower risk of postoperative delirium; however, a few cases suggest that it may also cause delirium-like neuropsychiatric reactions in certain patients. Although reserpine is used less frequently, the changes in central neurotransmitters after long-term exposure and recent discontinuation remain a concern. At present, there are no reports of acute behavioral abnormalities in patients who received dexmedetomidine sedation perioperatively after recent discontinuation of long-term reserpine treatment.

**Case presentation:**

A 63-year-old hypertensive woman, who had been taking reserpine for 10 years and discontinued it 7 days prior to surgery, underwent arthroscopic meniscectomy under subarachnoid anesthesia. After satisfactory anesthesia, she was given intravenous dexmedetomidine for stress sedation. During the infusion, the patient suddenly experienced disorientation, incoherent speech, significant resistance, and agitation, accompanied by a further increase in blood pressure. Immediately after an intravenous bolus of remimazolam 2 mg, the symptoms rapidly subsided, the patient returned to a sedated state, and the surgery was successfully completed. Postoperatively, the patient was conscious, did not exhibit similar abnormal behavior, had no recollection of the episode, and was discharged smoothly on the 3rd postoperative day.

**Conclusion:**

This case suggests that rare acute hyperactive delirium-like episodes may occur in patients who have recently discontinued long-term reserpine therapy and are receiving dexmedetomidine sedation during the perioperative period. Clinically, careful titration and close monitoring of mental status and hemodynamics are necessary.

## Background

Postoperative delirium (POD) is a common perioperative neurocognitive complication in older adults and is closely associated with adverse outcomes, including progressive cognitive decline, loss of functional independence, and increased healthcare burden. Identifying potentially modifiable drug-related factors is therefore of clear clinical importance (1). Dexmedetomidine, a highly selective α2-adrenoceptor agonist, is widely used for intraoperative and procedural sedation and is generally regarded as a relatively delirium-sparing sedative. Recent evidence supports this view. A 2024 meta-analysis of randomized controlled trials in non-cardiac surgery showed that intraoperative intravenous dexmedetomidine was associated with a lower incidence of POD, although bradycardia and hypotension occurred more frequently (2). In addition, among older patients undergoing lower-limb orthopedic surgery under spinal anesthesia, dexmedetomidine sedation was associated with a lower rate of POD than propofol sedation (3). Nevertheless, dexmedetomidine may not be uniformly protective in all settings, and recent case reports have described paradoxical delirium-like reactions during routine infusion (4).

Although reserpine is no longer a first-line antihypertensive drug, it is still used in some clinical situations. Existing research suggests that reserpine can alter central monoaminergic signaling by affecting the vesicular storage of monoamine neurotransmitters and may be associated with neuropsychiatric abnormalities (5, 6). However, relevant modern clinical data are very limited, and existing direct evidence mainly comes from early case reports on withdrawal psychosis or manic-like reactions (6, 7). To the best of our knowledge, no prior case report has specifically described this clinical scenario; therefore, we report an acute hyperactive delirium-like episode during dexmedetomidine sedation in a patient who had recently discontinued long-term reserpine therapy before surgery.

## Case presentation

A 63-year-old woman (height 160 cm, weight 55 kg) was scheduled to undergo arthroscopic meniscectomy of the knee. She had a 10-year history of hypertension treated with reserpine (0.1 mg/day), which had been discontinued 7 days before surgery. After discontinuation, her preoperative blood pressure remained relatively stable, ranging from 131/84 to 142/86 mmHg, and no alternative antihypertensive therapy was initiated before surgery. Her medical history was otherwise notable only for an appendectomy 20 years earlier. Preoperatively, cognitive function was normal, with no evidence of anxiety, sleep disturbance, or previous psychiatric illness. Laboratory findings were unremarkable.

In the operating room, standard monitoring was established, including invasive arterial blood pressure, electrocardiography, and pulse oxygen saturation (SpO₂). Her initial blood pressure was 175/98 mmHg, heart rate 75 beats/min, and SpO_2_ 98%. She was placed in the lateral position. After skin disinfection and sterile draping, local infiltration anesthesia was performed at the L3–4 interspace with 2 mL of 1% lidocaine. Spinal anesthesia was then performed using a 25-gauge spinal needle. After free flow of cerebrospinal fluid was confirmed, 12 mg of bupivacaine mixed with 0.3 mL of 10% glucose was injected to prepare a hyperbaric solution. The patient was then returned to the supine position, and the sensory block level was assessed as T10. The head of the operating table was elevated by approximately 30°. At that time, blood pressure increased to 205/104 mmHg, while heart rate was 78 beats/min and SpO_2_ was 100%. Dexmedetomidine was prepared at a concentration of 4 μg/mL. A loading dose of 0.5 μg/kg over 10 min was planned.

During skin preparation of both lower limbs, after approximately 20 μg of dexmedetomidine had been infused, the patient developed abrupt psychomotor agitation with manic-like behavior. She remained conscious but became clearly disoriented, repeatedly stating that she did not want the operation and wanted to go home. Her speech became incoherent, she was unable to follow instructions, reassurance was ineffective, and she showed marked resistance. At the same time, blood pressure rose further to 215/110 mmHg, with a heart rate of 82 beats/min and SpO₂ of 100%. Remimazolam 2 mg was administered intravenously immediately. The patient then became sedated, stopped speaking, and her blood pressure decreased to 161/86 mmHg. Arterial blood gas analysis, performed while the patient was receiving supplemental oxygen at 2 L/min, showed the following values: pH 7.39, PaCO₂ 53 mmHg, PaO₂ 80.7 mmHg, sodium 144 mmol/L, potassium 3.4 mmol/L, ionized calcium 1.19 mmol/L, blood glucose 7.8 mmol/L, and lactate 0.7 mmol/L.

No additional sedative or anesthesia-related drugs were administered thereafter, and the operation proceeded as planned. Sedation remained stable throughout surgery, which lasted approximately 70 min. At the end of the procedure, the patient opened her eyes when called and was able to communicate normally. She reported no recollection of the intraoperative episode and complained only of mild nausea without vomiting. At that time, her vital signs were stable: blood pressure 158/88 mmHg, heart rate 67 beats/min, and SpO₂ 100%. On postoperative day 1, she was in good condition with stable vital signs and no recurrence of abnormal neuropsychiatric symptoms. She was discharged uneventfully on postoperative day 3.

## Discussion and conclusions

In this case, the abrupt onset of disorientation, incoherent speech, inability to follow instructions, marked resistance, psychomotor agitation, and subsequent amnesia during dexmedetomidine sedation indicated a transient disturbance of cognition, communication, behavior, and memory. Although no standardized delirium assessment was performed, this clinical pattern was more compatible with an acute hyperactive delirium-like episode than with isolated intraoperative agitation (8). Acute hypertensive encephalopathy should also be considered in the differential diagnosis, because it may present with acute mental status changes and clinically overlaps with posterior reversible encephalopathy syndrome (PRES) (9, 10). Given the marked intraoperative blood pressure elevation, hypertension may have contributed to the episode and cannot be definitively excluded. However, the close temporal relationship with dexmedetomidine loading and the rapid resolution after sedative rescue without postoperative recurrence suggest that hypertension alone may not fully explain the clinical course. Mild hypercapnia was another possible contributor, as elevated PaCO₂ may affect mental status (11). However, because arterial blood gas analysis was performed after sedative rescue and showed normal pH with preserved oxygenation, hypercapnia alone was unlikely to explain the overall clinical course. A recent systematic review of medication-related delirium listed reserpine among cardiovascular drugs potentially associated with delirium risk and noted that it may be accompanied by central nervous system manifestations such as memory impairment and inattention (12). Reserpine is a lipophilic monoamine-depleting agent that can penetrate the central nervous system and inhibit vesicular monoamine transporters, leading to prolonged depletion of norepinephrine, dopamine, and serotonin (7). Given the non-selective role of VMAT2 in monoamine storage, serotonergic dysregulation may also have contributed to the patient’s neurochemical vulnerability, although no serotonin-related biomarkers were available in this case (5). Therefore, we do not propose a simple rebound increase in monoamine availability or an acute withdrawal hyperexcitability state 7 days after discontinuation. Rather, prolonged monoamine depletion, together with possible compensatory receptor- or network-level adaptations, may have affected neural processes involved in arousal, attention, and cognitive integration (13). Earlier case reports by Kent and Wilber and by Samuels and Taylor further described hallucinations, mania, and other withdrawal-related psychiatric symptoms following abrupt discontinuation of reserpine after long-term use, which may be related to compensatory receptor-level neuroadaptation, such as dopaminergic supersensitivity (14, 15). In summary, these observations suggest that recent discontinuation after long-term reserpine exposure may have represented a possible predisposing vulnerability in this patient, rather than a proven causal mechanism.

Dexmedetomidine is more likely a direct triggering factor, although multiple randomized trials and systematic reviews have shown that dexmedetomidine generally reduces the risk of delirium (16). Recent case reports and clinical studies have shown that delirium or delirium-like neuropsychiatric adverse reactions may still occur in susceptible patients or in specific clinical situations (4, 17, 18). In addition, dexmedetomidine may cause transient hypertension during the loading phase by activating peripheral α2-adrenergic receptors. In this case, the patient developed significant agitation and a further increase in blood pressure after infusion of approximately 20 μg of dexmedetomidine, which can be reasonably explained.

Delirium is essentially an acute brain dysfunction caused by the interaction of predisposing and precipitating factors (19). The underlying mechanisms of dexmedetomidine-related delirium or delirium-like neuropsychiatric responses are not fully elucidated. Existing evidence suggests that disruption of the locus coeruleus-norepinephrine arousal network may be involved. Dexmedetomidine inhibits locus coeruleus-related norepinephrine activity through central α2-adrenergic receptor agonism, and the norepinephrine system is essential for maintaining arousal, attention, and cognitive integration. In susceptible individuals, excessive inhibition or unstable rebound activity of this system could theoretically induce acute brain dysfunction (20–22). Therefore, a plausible explanation for this case is that discontinuation of reserpine caused monoaminergic vulnerability, while the central and peripheral effects of dexmedetomidine during the loading period played a direct precipitating role, together triggering an acute hyperactive delirium-like episode under perioperative stress.

The limitations of our study are as follows: First, standard delirium assessment tools were not used at the time of the event; therefore, the term “delirium-like episode” should be interpreted as a clinical description rather than a definitive diagnosis. Second, because no neuroimaging, fundoscopic examination, or formal neurological consultation was performed during the brief intraoperative episode, acute hypertensive encephalopathy or posterior reversible encephalopathy syndrome could not be definitively excluded. Third, arterial blood gas analysis was performed after sedative rescue rather than immediately before symptom onset; therefore, the temporal relationship between mild hypercapnia and the neuropsychiatric episode could not be determined. Finally, as a single case observation without drug concentration measurements, monoamine-related biomarkers, or serotonin-related assessments, this report can only suggest temporal associations and possible contributing factors, but cannot establish causality or confirm a synergistic interaction between reserpine discontinuation and dexmedetomidine.

In conclusion, dexmedetomidine should be used with caution in patients with a long history of reserpine use who have recently discontinued it, rapid loading doses should be avoided whenever possible, and their mental status and hemodynamic changes should be closely monitored.

## Data Availability

The raw data supporting the conclusions of this article will be made available by the authors, without undue reservation.

## References

[1] Thedim M Vacas S. Postoperative delirium and the older adult: untangling the confusion. J Neurosurg Anesthesiol. (2024) 36:184–9. doi: 10.1097/ANA.0000000000000971, 38683185 PMC11345733

[2] Wang D Liu Z Zhang W Zu G Tao H Bi C. Intravenous infusion of dexmedetomidine during the surgery to prevent postoperative delirium and postoperative cognitive dysfunction undergoing non-cardiac surgery: a meta-analysis of randomized controlled trials. Eur J Med Res. (2024) 29:239. doi: 10.1186/s40001-024-01838-z, 38637853 PMC11025279

[3] Shin H-J Woo Nam S Kim H Yim S Han S-H Hwang J-W . Postoperative delirium after dexmedetomidine versus propofol sedation in healthy older adults undergoing orthopedic lower limb surgery with spinal anesthesia: a randomized controlled trial. Anesthesiology. (2023) 138:164–71. doi: 10.1097/ALN.0000000000004438, 36534899

[4] Sun L Mu J Wang Y He H. Perioperative dexmedetomidine-induced delirium in a patient with schizophrenia: a case report. BMC Anesthesiol. (2024) 24:278–83. doi: 10.1186/s12871-024-02670-y, 39123151 PMC11312422

[5] Wang Y Zhang P Chao Y Zhu Z Yang C Zhou Z . Transport and inhibition mechanism for VMAT2-mediated synaptic vesicle loading of monoamines. Cell Res. (2024) 34:47–57. doi: 10.1038/s41422-023-00906-z, 38163846 PMC10770148

[6] Strawbridge R Javed RR Cave J Jauhar S Young AH. The effects of reserpine on depression: a systematic review. J Psychopharmacol. (2023) 37:248–60. doi: 10.1177/02698811221115762, 36000248 PMC10076328

[7] Carnovale C Perrotta C Baldelli S Cattaneo D Montrasio C Barbieri SS . Antihypertensive drugs and brain function: mechanisms underlying therapeutically beneficial and harmful neuropsychiatric effects. Cardiovasc Res. (2023) 119:647–67. doi: 10.1093/cvr/cvac110, 35895876 PMC10153433

[8] Ahmadzadeh S Duplechin DP Haynes AT Hollander AV Rieger R Jean Baptiste C . Evolving clinical management of postoperative delirium: a narrative review. Cureus. (2025) 17:e92927. doi: 10.7759/cureus.92927, 41133074 PMC12542918

[9] Fugate JE Rabinstein AA. Posterior reversible encephalopathy syndrome: clinical and radiological manifestations, pathophysiology, and outstanding questions. Lancet Neurol. (2015) 14:914–25. doi: 10.1016/S1474-4422(15)00111-8, 26184985

[10] Miller JB Suchdev K Jayaprakash N Hrabec D Sood A Sharma S . New developments in hypertensive encephalopathy. Curr Hypertens Rep. (2018) 20:13–9. doi: 10.1007/s11906-018-0813-y, 29480370

[11] Davidson AC Banham S Elliott M Kennedy D Gelder C Glossop A . BTS/ICS guideline for the ventilatory management of acute hypercapnic respiratory failure in adults. Thorax. (2016) 71:ii1–ii35. doi: 10.1136/thoraxjnl-2015-208209, 26976648

[12] Weidmann AE Matthíasdóttir R Proppé GB Tadić I Gunnarsson PS Jónsdóttir F. Medication causes and treatment of delirium in patients with and without dementia. Brain Behav. (2025) 15:e70706. doi: 10.1002/brb3.70706, 40686036 PMC12277658

[13] Bernstein AI Stout KA Miller GW. The vesicular monoamine transporter 2: an underexplored pharmacological target. Neurochem Int. (2014) 73:89–97. doi: 10.1016/j.neuint.2013.12.003, 24398404 PMC5028832

[14] Samuels AH Taylor AJ. Reserpine withdrawal psychosis. Aust N Z J Psychiatry. (1989) 23:129–30. doi: 10.3109/00048678909062604, 2930412

[15] Kent TA Wilber RD. Reserpine withdrawal psychosis: the possible role of denervation supersensitivity of receptors. J Nerv Ment Dis. (1982) 170:502–4. doi: 10.1097/00005053-198208000-00011, 7097268

[16] Luney M Holdsworth L Hagana A Kaminskaite V Qiu L Berner J . Effectiveness of drug interventions to prevent delirium after surgery for older adults: systematic review and network meta-analysis of randomised controlled trials. BMJ. (2026) 392:e085539. doi: 10.1136/bmj-2025-085539, 41690769 PMC12895303

[17] Shuai Y Liu Y Yang X Wan Q Zhao J Wang X. Delirium risk associated with esketamine, sevoflurane, propofol, and dexmedetomidine: a real-world study based on the FDA adverse event reporting system. Eur J Pharmacol. (2025) 1000:177723. doi: 10.1016/j.ejphar.2025.177723, 40348319

[18] Andrei SE Zhuo W Shiekh KN Reinert JP. Effect of dexmedetomidine on rescue analgesic needs in non-intubated intensive care patients. J Pharm Technol. (2025) 41:18–21. doi: 10.1177/87551225241288137, 39545245 PMC11559726

[19] Wilson JE Mart MF Cunningham C Shehabi Y Girard TD MacLullich AMJ . Delirium. Nat Rev Dis Primers. (2020) 6:90. doi: 10.1038/s41572-020-00223-4 33184265 PMC9012267

[20] Nelson LE Lu J Guo T Saper CB Franks NP Maze M. The alpha2-adrenoceptor agonist dexmedetomidine converges on an endogenous sleep-promoting pathway to exert its sedative effects. Anesthesiology. (2003) 98:428–36. doi: 10.1097/00000542-200302000-00024, 12552203

[21] Kang R Jeong JS Ko JS Lee S-Y Lee JH Choi SJ . Intraoperative dexmedetomidine attenuates norepinephrine levels in patients undergoing transsphenoidal surgery: a randomized, placebo-controlled trial. BMC Anesthesiol. (2020) 20:100. doi: 10.1186/s12871-020-01025-7, 32359367 PMC7195722

[22] Hansen N Rediske AI. The locus coeruleus noradrenaline system in delirium. Front Aging Neurosci. (2021) 13:784356. doi: 10.3389/fnagi.2021.784356, 34955815 PMC8692941

